# Current state and future of co-inhibitory immune checkpoints for the treatment of glioblastoma

**DOI:** 10.20892/j.issn.2095-3941.2020.0027

**Published:** 2020-08-15

**Authors:** Shaoping Shen, Ling Chen, Jialin Liu, Lin Yang, Mengna Zhang, Lingxiong Wang, Rong Zhang, Yasushi Uemura, Qiyan Wu, Xinguang Yu, Tianyi Liu

**Affiliations:** ^1^Department of Neurosurgery, Chinese PLA General Hospital, Beijing 100853, China; ^2^Pediatric Center, Chinese PLA General Hospital, Beijing 100853, China; ^3^Key Laboratory of Cancer Center, Chinese PLA General Hospital, Beijing 100853, China; ^4^Division of Cancer Immunotherapy, Exploratory Oncology Research and Clinical Trial Center, National Cancer Center, Kashiwa 277-8577, Japan

**Keywords:** Immunotherapy, glioblastoma, co-inhibitory immune checkpoint, checkpoint inhibitors, combination therapy

## Abstract

In the interaction between a tumor and the immune system, immune checkpoints play an important role, and in tumor immune escape, co-inhibitory immune checkpoints are important. Immune checkpoint inhibitors (ICIs) can enhance the immune system’s killing effect on tumors. To date, impressive progress has been made in a variety of tumor treatments; PD1/PDL1 and CTLA4 inhibitors have been approved for clinical use in some tumors. However, glioblastoma (GBM) still lacks an effective treatment. Recently, a phase III clinical trial using nivolumab to treat recurrent GBM showed no significant improvement in overall survival compared to bevacizumab. Therefore, the use of immune checkpoints in the treatment of GBM still faces many challenges. First, to clarify the mechanism of action, how different immune checkpoints play roles in tumor escape needs to be determined; which biomarkers predict a benefit from ICIs treatment and the therapeutic implications for GBM based on experiences in other tumors also need to be determined. Second, to optimize combination therapies, how different types of immune checkpoints are selected for combined application and whether combinations with targeted agents or other immunotherapies exhibit increased efficacy need to be addressed. All of these concerns require extensive basic research and clinical trials. In this study, we reviewed existing knowledge with respect to the issues mentioned above and the progress made in treatments, summarized the state of ICIs in preclinical studies and clinical trials involving GBM, and speculated on the therapeutic prospects of ICIs in the treatment of GBM.

## Introduction

Glioblastoma (GBM) is the most common and aggressive primary malignant brain tumor, and is associated with an extremely poor prognosis and a median survival time of only 8–12 months^1–4^, with a 5-year survival rate still less than 10%^5^. The current standard of care (SOC) for GBM is maximal surgical resection followed by radiotherapy and temozolomide chemotherapy, and to date, no other drugs have been added to the SOC. Targeted agents and antiangiogenic therapy have failed to show survival benefits in randomized clinical trials^6^. Therefore, novel treatment strategies are urgently needed.

Cancer immunotherapies, which boost nonspecific innate or tumor-specific adaptive immunity, have recently been extensively used in modern oncology. Immunotherapies have exhibited unprecedented efficacy in the treatment of some solid tumors, and among immunotherapeutic approaches, immune checkpoint inhibitors (ICIs) were researched relatively early and have produced dramatic changes in the treatment paradigms of a number of challenging cancers, including metastatic melanoma^7^, non-small cell lung cancer (NSCLC)^8–10^, renal cell carcinoma (RCC)^11^, and bladder carcinoma^12^, so they may also provide a new direction for the treatment of GBM.

**Figure 1:**
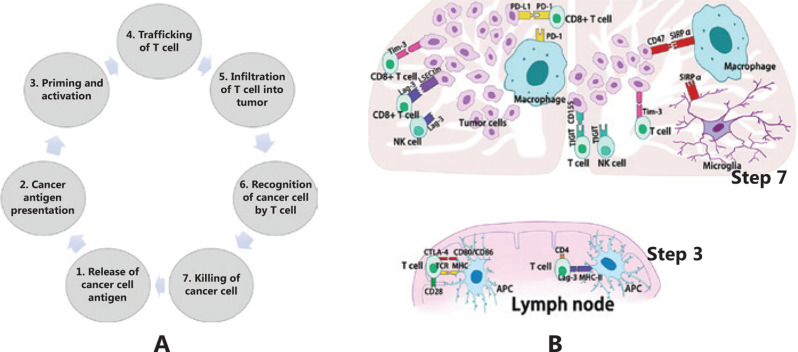
A. Cancer-immunity cycle. The cycle includes seven steps: 1, cancer cell antigen release; 2, cancer antigen presentation; 3, priming and activation; 4, trafficking of T cells; 5, infiltration of T cells into tumors; 6, recognition of cancer cells by T cells; and 7, killing of cancer cells. Immune checkpoint pathways play roles in antigen priming/activation of T cells (step three) and killing of cancer cells (step seven). B. In glioblastoma immunity, co-inhibitory immune checkpoints mainly play roles in antigen priming/activation of T cells (in lymph nodes) and killing of cancer cells (in the tumor microenvironment). In lymph nodes, CTLA-4 and LAG-3 can compete with the receptor-ligand binding with antigen-presenting cells, which leads to decreased T cell activation and responsiveness. In the tumor microenvironment, PDL1, Tim-3, LAG-3, TIGIT, and SIRP can bind with their ligands in effector cells, including T cells, NK cells, and macrophages, to consequently influence their efficiency in tumor killing.

**Figure 2:**
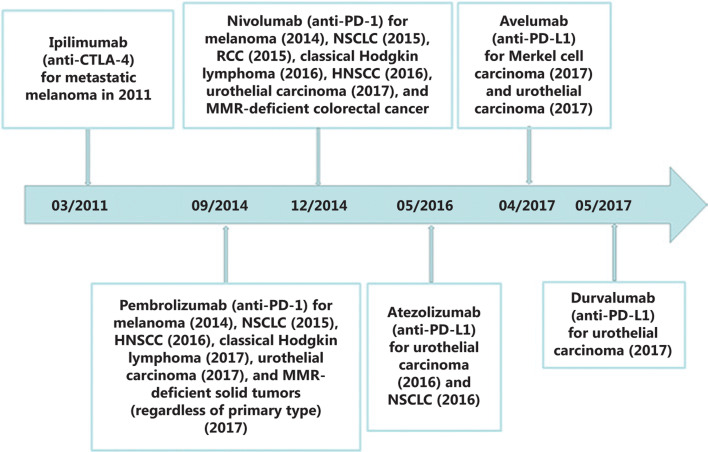
Cytotoxic T lymphocyte-associated protein 4 (CTLA4) and programmed death 1 (PD1) are two well-studied immune checkpoint molecules, and currently, they can both be targeted by humanized antibodies that have been approved by the U.S. Food and Drug Administration (FDA), for clinical use.

In the antitumor immune response, we expect that the immune system automatically rejects cancer cells as foreign based on the unique and often extensive mutational profiles of cancer cells. However, in practical situations, there is a natural balance between the immune system and cancer, which is called adaptive immune tolerance, and it is maintained by multiple mechanisms, including immune checkpoint pathways. Normally, these pathways play critical roles in the maintenance of immune homeostasis, and this function can be induced by cancer cells to evade immune attack. According to the cancer-immunity cycle^13^ (**Figure 1A**), these pathways mainly play roles in antigen priming/activation of T cells (step three) and killing of cancer cells (step seven) (**Figure 1**). The checkpoint pathways include costimulatory signals that combat tumor growth and co-inhibitory signals that promote tumor growth *via* the immune response; at present, most studies are focused on co-inhibitory pathways for cancer treatment. Physiologically, through the binding of receptors and their ligands, these signals can attenuate autoimmunity by inhibiting cytotoxic T cell functions and reducing the proliferation of these cells, while in cancer immunology, these signals play an important role in helping the tumor evade the immune system. To date, several ICIs have been investigated. Cytotoxic T lymphocyte-associated protein 4 (CTLA4) and programmed death 1 (PD1) are the two best-studied immune checkpoint molecules, and currently, they can both be targeted by humanized antibodies that have been approved by the U.S. Food and Drug Administration (FDA) for clinical use (**Figure 2**); these antibodies have exhibited unprecedented efficacy in several cancer indications. In general, humanized antibodies used as ICIs alleviate immunosuppression by binding to either a ligand or receptor. Other co-inhibitory immune checkpoint molecules have been identified, such as lymphocyte activation gene-3 (LAG-3), T-cell immunoglobulin, and mucin-3 (Tim-3), and T-cell immunoglobulin and the ITIM domain (TIGIT), which differ from each other in many ways (**Figure 1B**).

The application of immunotherapy in the treatment of intracranial tumors started late. Initially, it was thought that the brain had no lymphatic system, which would make the brain an immune-privileged organ^14,15^. However, researchers have found that the brain is monitored by the immune system and that a lymphatic system that communicates with the extracranial lymphatic system exists^16,17^. These findings provide an anatomical basis for immunotherapy of intracranial tumors. Therefore, many exploratory studies have been performed on immunotherapy for GBM, and ICIs are of great interest. Studies have shown that PDL1 is highly expressed on GBM cells^18,19^, and combinational checkpoint blockade immunotherapy has demonstrated promising efficacy in preclinical GBM mouse models. However, checkpoint blockade has not yet resulted in breakthroughs in GBM clinical trials similar to those in clinical trials of other tumors. The reason may be that the PD1/PDL1 pathway only plays a role in the malignant biological behavior of GBM, while other molecular signaling networks also play indispensable roles. Other reasons, including tumor genetic characteristics, the tumor microenvironment (TME), and decreased numbers of infiltrating lymphocytes, may lead to poor effects. Therefore, in GBM therapy, issues including how to select an ICI, which ICI to select, and how to make decisions regarding combination therapies to improve therapeutic efficacy need to be studied further to provide specific guidance for the development of GBM immunotherapy clinical trials.

## Mechanism of action of ICIs and their application in tumor immunotherapy

### PD1/PDL1

PD1 is expressed on activated T cells, B cells, natural killer (NK) cells, and myeloid cells. PD1 expression is often upregulated in the TME, while its ligands, including PDL1 (CD274, B7-H1) and PDL2 (CD273, B7-DC), are upregulated in activated leukocytes and myeloid cells as well as in many cancer cells. In the TME, cancer cells and myeloid cells are thought to be the main cell types mediating T cell suppression through the PD1 pathway^20,21^. Thus, anti-PD1/PDL1 blocking antibodies are thought to act predominantly within tumors.

It has been reported that the durable objective (partial or complete) response rate following anti-PD1 therapy is 31%–44% in patients with advanced melanoma^7,22–25^, 19%–20% in patients with NSCLC^8–10,26^, and 22%–25% in patients with RCC^11,27^, and the overall survival (OS) is also extended by PD1 therapy compared with conventional therapies. To date, five antibodies that target the PD1/PDL1 axis have been approved by the FDA (**Figure 2**) for advanced or unresectable melanoma and NSCLC, and pembrolizumab (an anti-PD1 antibody) induces a better response than ipilimumab (an anti-CTLA4 antibody)^24^. Anti-PD1 therapy has also shown some efficacy in head and neck, breast, ovarian, and gastric cancers.

### CTLA4

CTLA4, a homolog of CD28 [a costimulatory factor of the T-cell receptor (TCR)], is expressed on T cells, and its ligands are CD80 and CD86, which are expressed on the surface of antigen-presenting cells (APCs)^28–30^. By competing with CD28 the costimulatory receptor for binding to their ligands, CTLA4 decreases T cell activation and responsiveness, although the precise mechanisms are not fully understood. In addition, CTLA4 is also constitutively expressed on regulatory T cells (Tregs), contributing to their immunosuppressive functions^29,31^. Thus, anti-CTLA4 blocking antibodies are thought to act predominantly within lymph nodes and work across a wider range than other antibodies.

According to previous research, 22% of advanced-stage melanoma patients treated with an anti-CTLA4 antibody have durable responses extending beyond 10 years^32^. Ipilimumab, an anti-CTLA4 antibody, was the first ICI to be approved by the FDA in 2011, and was approved for the treatment of metastatic melanoma. It has also been tested in other malignancies, including NSCLC, renal cancer, and prostate cancer; however, these trials did not meet the projected clinical endpoints^33^. Unlike other ICIs, anti-CTLA4 blocking antibodies predominantly function in T cell priming and activation, and they enhance the immunosuppressive activity of Tregs. Thus, CTLA4 blockade has a global impact on the immune system; therefore, with the advent of other specific inhibitors, its clinical use has gradually decreased.

Generally, CTLA4 and PD1/PDL1 are regarded as the first tier of co-inhibitory checkpoint molecules, which are primarily responsible for maintaining self-tolerance, and LAG-3, Tim-3, and TIGIT are regarded as representative of the second tier of co-inhibitory molecules, which have distinct and more specific roles in regulating the immune response^34^; these second-tier molecules may also have different lymphoid, anatomical, and functional specifications. There have been some preclinical studies and clinical trials assessing their functions.

### LAG-3

LAG-3 (CD223) is mainly expressed on the surface of B cells, NK cells, tumor-infiltrating lymphocytes (TILs), and a subset of T cells. In CD4+ T cells, LAG-3 is a CD4 homolog, has a higher affinity than CD4 for MHC-II, and inhibits TCR-induced calcium ion fluxes, compromising CD4+ T cell activation. In terms of CD8+ T lymphocytes and NK cells, LAG-3 does not work through MHC-II but rather works through LSECtin, another ligand of LAG-3, which is mainly expressed on tumor cells. Therefore, LAG-3 plays roles not only in the TME but also in the early stage of T cell activation^35^.

In preclinical studies of murine models of cancer, LAG-3 and PD1 have been shown to be co-expressed on both CD4+ and CD8+ TILs, and co-blockade of the Lag-3 and PD1 pathways has been shown to synergize to improve antitumor CD8+ T cell responses^36^. LAG-3 blockade has also been shown to synergize with antitumor vaccination to improve tumor-specific CD8+ T cell activation. In some early phase I/II clinical trials, soluble LAG-3-Ig IMP321 was used to treat advanced RCC (tumor shrinkage)^37^, advanced pancreatic adenocarcinoma (combined with chemotherapy but lacked activity with suboptimal dosing)^38^, advanced melanoma (combined with MART-1 peptide vaccination, which showed an increase in CD8+ T cell numbers and a decrease in Treg numbers)^39^, and metastatic breast carcinoma (phase I/II; combined with chemotherapy, which showed an objective response rate of 50%)^40^. Although positive responses were not observed per response evaluation criteria in solid tumor RECIST criteria, some efficacy has been shown in the clinic. Recently, antibodies that block LAG-3 binding to MHC-II have been used in the clinic, and the use of anti-LAG-3 antibodies either alone or in combination with anti-PD1 antibodies is being used in both solid and hematological tumors^35^.

### Tim-3

Tim-3 was initially identified as a cell-surface molecule selectively expressed on IFN-γ-producing CD4+ T helper 1 (Th1) and CD8+ T cytotoxic 1 (Tc1) cells. Tim-3 has recently been identified on Tregs and innate immune cells [dendritic cells (DCs), NK cells, and monocytes]. Humans have three Tim genes: *Havcr1* (Tim-1), *Havcr2* (Tim-3), and *Timd4* (Tim-4). The ligands of Tim-3 include C-type galectin-9, PtdSer, HMGB1, and CEACAM1, and through binding with different ligands, Tim-3 plays different roles in innate and adaptive immune responses. Thus, Tim-3 acts on both innate and adaptive immunities and is thought to be an important regulator of CD8+ T cell exhaustion in cancer^41^.

There have been some preclinical studies of anti-Tim-3 antibodies acting on models of solid or hematological tumors. In clinical trials, Tim-3 expression is considered a marker of dysfunctional/exhausted T cells, and Tim-3 blockade improves the function of these cells, especially when combined with PD1 co-blockade, showing a relatively strong effect^42,43^ on patients with advanced metastatic melanoma^42^, NSCLC^44^, or follicular B-cell non-Hodgkin lymphoma (FL)^43^.

### TIGIT

TIGIT is a member of the CD28 family and is expressed on NK cells, activated and memory T cells, and subsets of Tregs and follicular T helper (Tfh) cells^45,46^. Its ligands, CD155 and CD112, are mainly expressed on APCs, T cells, and a variety of nonhematopoietic cell types, including tumor cells^35^. Multiple groups^45,47–49^ have shown that TIGIT contributes to immunotolerance by inhibiting immune responses mediated not only by T cells but also by NK cells through binding of its CD155 ligand on APCs or target cells.

Similar to the results for Tim-3 and LAG-3, previous findings have indicated that co-blockade of TIGIT and PD1 additively improved CD8+ TIL proliferation, cytokine production, and degranulation in melanoma patients. In addition, TIGIT synergizes not only with PD1 but also with Tim-3 to impair protective antitumor responses^50^. In addition to the direct suppression of CD8+ TILs, indirect suppression *via* the promotion of Tregs can also suppress antitumor immunity.

### CD47-SIRPα

The CD47-SIRPα signaling pathway is different from the other pathways previously mentioned. This phagocytosis-related checkpoint molecule is mainly expressed on macrophages and other innate immune cells. The signal-regulatory protein (SIRP) family encompasses five members with varying levels of amino acid sequence homology, including SIRPα, SIRPβ1, SIRPγ, SIRPβ2, and SIRPδ, and among them, SIRPα is the most thoroughly studied member. It is an inhibitory receptor expressed on myeloid cells, including macrophages, monocytes, DCs, and neutrophils^51–57^, and it is also expressed at varying levels on neuronal cells in the central nervous system (CNS); most of these cells promote adaptive T cell-mediated immunity against cancer. Its ligand, the “don’t eat me” signal CD47, is broadly expressed on the plasma membrane of essentially all cell types and is often overexpressed on cancer cells. Blocking the CD47-SIRPα interaction has been shown to promote the destruction of cancer cells by phagocytes, including macrophages and neutrophils. Targeted antibodies, such as anti-CD47 antibodies, engineered receptor decoys, anti-SIRPa antibodies, and bispecific agents have been developed and are now under preclinical and clinical investigations.

Numerous studies have shown that tumor-associated macrophages (TAMs) have dual supportive and inhibitory influences on cancer, depending on the disease stage, the tissue involved, and the host microbiota^58^. Previously, immunotherapies targeting TAMs mainly focused on macrophage depletion, which provided a survival advantage in several types of cancers. Recently, therapeutic strategies have switched to activating and re-educating macrophages. Therapies targeting the CD47/SIRPa axis belong to this latter strategy and have demonstrated success in a wide range of preclinical models (including acute myeloid leukemia, non-Hodgkin lymphoma, acute lymphocytic leukemia, myeloma, ovarian cancer, colon cancer, breast cancer, and bladder cancer)^59–61^; they are now under investigation in clinical trials for both solid and hematological malignancies. To date, several phase I clinical trials have been conducted. Recently, Advani et al.^62^ reported the results of their phase Ib clinical trial using Hu5F9-G4 (an ICI blocking CD47) in B-cell non-Hodgkin lymphoma patients. A total of 22 patients were enrolled, and 95% of them were refractory to rituximab. The results showed that 50% of the patients had an objective (i.e., complete or partial) response, with 36% having a complete response. The objective response and complete response rates were 40% and 33%, respectively, and the macrophage checkpoint inhibitor 5F9 combined with rituximab showed promising efficacy in patients with aggressive and indolent lymphoma. The authors concluded that the higher the myeloid cell number in the TME, the better the effect of this treatment.

## Application of ICIs in GBM

### PD1/PDL1

Compared with studies in other tumors, studies of immunotherapy in brain tumors started relatively late due to the difficulties associated with recruiting immune cells into the brain. With the discovery of the brain immune system, immunotherapies including ICIs for GBM were rapidly developed. Accordingly, PD1/PDL1 inhibitors are currently the most widely researched ICIs in GBM as a result of their safety and effectiveness. Currently, more than 30 clinical trials have been performed^63,64^. Some of these trials have finished and have available data (NCT02017717, NCT02336165, NCT02054806, and NCT02313272)^64^. The results of phase I/II trials have confirmed the safety and tolerability of PD1/PDL1 inhibitors in GBM treatment. However, the only phase III result (checkmate 143) showed that compared with bevacizumab, nivolumab (an anti-PD1 antibody) did not improve the progression-free survival (PFS) or OS in recurrent GBM^65^. Recently, a randomized, multi-institutional clinical trial of neoadjuvant pembrolizumab was conducted by the Ivy Consortium in 35 patients with recurrent surgically resectable GBM to evaluate immune responses and subsequent survival^66^. The results showed that neoadjuvant pembrolizumab conferred significant improvement in the patients’ OS and PFS and was associated with the upregulation of T cell and interferon-γ-related gene expression and downregulation of cell cycle-related gene expression within the tumor. Although an improvement in survival was observed, the underlying mechanism was not clear. The exact reason for the variability of the responses of anti-PD1 antibodies is still unknown, although we may be able to explore the underlying mechanism using the experience gained with other tumors.

In the treatment of other tumors, some biomarkers associated with the efficacy of anti-PD1/PDL1 therapy have been found; and a review by Suzanne L. Topalian^67^ provides a systematic summary based on immunological, genetic, and virological criteria. (1) Immunological biomarkers include intratumoural lymphoid infiltrates; intratumoural PDL1 expression upregulation regardless of whether it is caused by a genetically driven mechanism or adaptive immune resistance; and dynamic immunohistochemical observations of PDL1. (2) Genetic biomarkers include oncogenic mutations, the tumor mutational burden (TMB), and DNA mismatch repair (MMR) deficiency in cancer cells, which may correlate with the response and resistance to PD1/PDL1 therapy. (3) The virological criteria include proteins from oncogenic viruses that may act as immunogenic neo-antigens, and stimulate endogenous antitumor immune responses. Recently, some studies have supplemented these criteria with results from different perspectives. In Havel’s review^68^, mechanistic underpinnings, including tumor genomes, patient germline genetics, the immune microenvironment, systemic markers and the commensal microbiota, were introduced in a more systematic and detailed way. This review suggested the necessity of developing a predictive model that can take into account the different components that affect tumor-host interactions.

Some biomarkers previously mentioned have been shown to be applicable to GBM. For example, patients with “hypermutant-GBM” (i.e., a pediatric-GBM “sub-type” with high mutational burden resulting from biallelic MMR deficiency) showed a promising result when treated with anti-PD1 antibody^69^. Unfortunately, in most cases, GBM, which does not usually possess a carcinogen-induced mutational signature, exhibited relatively low TMB^70^ and displayed one of the lowest predicted neo-antigen burdens. The data showed that only 3.5% of GBM had a high TMB^71^. Even the association of MMR genes (including *MLH1*, *MSH2*, *MSH6*, and *PMS2*) with the efficacy of immunotherapy remains to be studied. However, some researchers have determined that MMR-induced mutations tend to be predominantly subclonal, which leads to highly heterogeneous tumors^72^ (intratumoural heterogeneity) and may elicit relatively ineffective antitumor immune responses^68^. Other types of mutations, such as those in POLE and POLD, which encode DNA polymerases and may cause genomic hypermutation, exist in some GBM specimens, and Hodges^71^ found that some of these mutations were associated with the highest TMBs. However, due to a lack of sufficient samples, their correlation with ICI therapy was unclear. For specific mutated genes for GBM immunotherapy, the research is scattered, and *MLH1*, *MSH2*, *MSH6*, *ATM*, and *PIK3CA* mutations are significantly associated with a high TMB. However, the exact influence of these mutations on immunotherapy is still unclear.

In addition to genetic biomarkers, other important biomarkers, such as intratumoral PDL1/PD1 expression and TILs infiltration, also failed to work. An analysis of *PDCO1* (codes for PD1) expression in the GBM/normal brain samples from The Cancer Genome Atlas and REMBRANDT data sets showed that there was no significant difference between GBM and normal brain samples^73^. As for intratumoral TIL infiltration, most data showed that GBM patients exhibited one of the lowest basal/preexisting TIL-associated genetic signatures among various solid tumor types. In contrast to other tumors, GBM has a completely different TME, which increases the complexity of GBM treatment by immunotherapy. According to the immunogenomic analysis of 33 diverse cancer types, performed by Thorsson^74^, GBM belongs to the lymphocyte-depleted type, which is characterized by a relatively prominent macrophage signature with the Th1 response suppressed and a high M2 response, and TIL numbers in GBM are lower than those in other tumor types^18^. GBM is not inherently immunogenic and is relatively unlikely to have a high density of CD8+ TILs. In newly diagnosed GBMs, the density of CD8+ TILs within the tumor tissue was described as being sparse in 50% of tumors and moderate in 7% of tumors in one study^18^. The probable reason was that standard radiation and temozolomide treatment diminished the potential pool of circulating tumor-reactive T cells^75^, and this depletion could be antagonistic to immunotherapy. In addition, tumor location in the CNS induces systemic immunosuppression and bone marrow suppression independent of histology due to the secretion of immunosuppressive cytokines by tumor-infiltrating myeloid cells^76^. GBM also exhibits increased Treg accumulation and elevated expression of TGF-beta.

In conclusion, the clinical biomarker analysis of GBM patients has delineated a low mutational/neoantigen burden, relatively low tumoral expression of immune checkpoints, and sparse pre-existing levels of TILs, which all indicate that adult GBM probably does not have an intrinsic predisposition toward therapies targeting immune checkpoints. Due to the complicated characteristics of GBM, there were limitations in the application of the biomarkers suitable for other tumors. Therefore, the correlation between GBM and the response to immunotherapy still requires additional data to obtain more comprehensive analyses. As investigations accumulate, we look forward to the development of a predictive model for GBM immunotherapy that takes into account different components, and dynamic data may systematically predict the therapeutic effects.

### CTLA4

Although CTLA4 was the first immunoregulatory molecule to be targeted for therapeutic purposes utilizing a humanized antibody, it was not widely adopted for clinical trials in GBM (**Table 1**). The reason may be the critical role of ipilimumab, which functions in the earlier phase of T cell activation and can cause an extensive impact on the immune network^68^. Some experiments have shown that cancer patients undergoing anti-PD1 immunotherapy experience less toxicity than patients treated with an anti-CTLA4 antibody^24^. Because the PD1 and CTLA4 signaling pathways are functionally nonredundant^77^, there are some clinical trials employing combination therapy targeting CTLA4 and PD1, and combination therapy has shown better results than monotherapies, although clinicians still need to consider these adverse events.

For different mechanisms of action of CTLA4 blockade, the biomarkers of response and resistance to anti-CTLA4 therapy differ from those for other ICIs^34^. Many studies have focused on the diversity, phenotype, and function of peripheral blood lymphocytes before and after therapy, and others have noted that a rise in the absolute lymphocyte count in the peripheral blood correlates with an increased rate of response to ipilimumab^78^. Other factors, such as high levels of soluble CD25^79^ (also known as IL2Rα) and elevated peripheral blood levels of a poorly differentiated population of myeloid cells^80–82^ [known as myeloid-derived suppressor cells (MDSCs)], have been reported to correlate with resistance to anti-CTLA4 therapy^79^. For local factors in the pretreatment TME, no clear predictive biomarkers have been identified for the selection of patients for CTLA4 blockade therapy.

Because most experiments performed to date have targeted CTLA4 or PD1/PDL1, it is reasonable to refer to these experiments when exploring other inhibitors and conducting further research.

As previously mentioned, LAG-3, Tim-3, and TIGIT are generally regarded as representative of the second tier of co-inhibitory molecules with distinct roles in regulating the immune response. Preclinical studies and clinical trials (**Table 2**) targeting these molecules have mostly used second-tier ICIs in combination with first-tier ICIs. Due to their mechanism of action, there are currently no validated biomarkers that predict which patients will benefit most from this dual blockade approach. As previously described, the inhibitory functions of LAG-3, Tim-3, and TIGIT may become evident only in susceptible backgrounds or upon active induction of disease, or these molecules may provide specificity to the regulation of immune responses in specific tissues *via* the expression of different co-inhibitory receptors on distinct lymphocyte subsets and the expression of the corresponding ligands in specific tissue sites. In addition, the Tim-3 and TIGIT pathways are believed to play dominant roles in regulating immune responses in the CNS^35^.

### LAG-3/Tim-3/TIGIT

LAG-3 is reported to be expressed in human GBM samples and a mouse GBM model. In preclinical studies, knocking out LAG-3 or inhibiting it with a blocking antibody is efficacious against GBM and can be used in combination with other ICIs to completely eradicate GBM-model tumors. With an anti-LAG-3 blocking antibody, early treatment is more efficacious than later treatment, possibly because LAG-3 is an early marker of T cell exhaustion. There are clinical trials underway targeting LAG-3 to treat GBM^64^.

In preclinical studies, the frequency of PD1+/Tim-3+ brain-infiltrating lymphocytes increased with time, and a Tim-3 inhibitor combined with PD1 blockade or stereotactic radiosurgery resulted in long-term survival^83^. Studies of Tim-3 expression in GBM specimens have demonstrated that the Tim-3 level is significantly elevated on both circulating blood lymphocytes and TILs in glioma patients. Tim-3 expression was positively correlated with glioma grade and negatively correlated with Karnofsky performance status score^84^. These findings indicate that Tim-3 is a potential clinical target for cancer therapy.

TIGIT is a novel checkpoint molecule recently discovered to play a role in cancer immunity^85–87^. Preclinical studies showed that TIGIT expression was upregulated on CD8+ T cells and Tregs in the brain^88,89^ compared to those in the draining cervical lymph nodes and spleen. In GBM patient samples, TIGIT expression was shown to be elevated on TILs, suggesting that the TIGIT pathway may be a promising immunotherapeutic target for the management of these patients^90^.

### The CD47-SIRPα pathway

The reason why the CD47-SIRPα pathway deserves attention in GBM patients is because the majority of immune cells within brain tumors are macrophages^91^, which often compose up to 30%–50% of the tumor mass and include tissue-resident microglia and bone marrow-derived monocytes and macrophages (BMDMs)^56,92^. TAMs have been reported to play very important roles in GBM progression, such as pro-tumorigenic roles *via* the release of cytokines, and they have been implicated in brain tumor angiogenesis and resistance to antiangiogenic therapies. Some preclinical experiments have shown that TAMs within the brain tend to be pro-tumorigenic^56,93^, and depletion strategies can produce a survival advantage in several types of cancer. However, considering the lymphatic constitution of the brain TME, modulation and reeducation of TAMs by enhancing the phagocytosis of glioma cells is considered a more promising antitumor strategy than depletion^94,95^. Some experiments have shown that in GBM, disruption of the SIRPα-CD47 signaling axis is an efficacious method of reeducating TAMs and enhancing tumor cell phagocytosis. To date, preclinical studies in mice have shown that CD47-SIRPα myeloid cell-directed checkpoint blockades effectively enhance tumor cell phagocytosis and thus reduce the tumor burden. Moreover, not only macrophages recruited from the periphery but also brain-resident microglia^96^ are effector cells that perform tumor cell phagocytosis in response to anti-CD47 blockade. Therefore, for brain tumors, blockade of the CD47-SIRPα pathway may generate promising effects.

## The future of combination treatment in GBM immunotherapy

Given the understanding of the mechanism underlying co-inhibitory immune checkpoint inhibition, it is explainable why ICIs are not effective against all cancer types or in every patient with a responsive type of cancer. Hence, the design of clinical trials and the application of immunotherapy should be more purposeful and rational. As Havel^68^ stated in his review, “Decisions regarding which immunotherapy to use or whether a combination approach is warranted should ideally be guided by rational mechanistic insight to maximize disease control, reduce side effects and minimize cost.”

According to the count and density of TILs within the tumor bed, GBM is classified as a “cold” tumor due to the lack of infiltrating T cells, so it may be difficult for ICI monotherapy to achieve efficacy. Therefore, researchers now widely consider how to “heat up” a tumor before ICI intervention. These strategies mainly focus on therapies that can increase the amount of TILs or reduce lymphocyte depletion. For this purpose, several treatments including vaccines, chimeric antigen receptor (CAR)-T cell therapy, and viral therapy have been developed.

GBM vaccines include direct exposure to antigens (peptide or DNA) and stimulated patient-derived APCs (DCs), both of which are designed to induce an immune response against the tumor. Modified GBM tumor lysates, such as those with heat shock proteins, combined with ICIs have been adopted for study in clinical trials (NCT03018288).

For patients who have surgically accessible disease, custom vaccines are a more promising option. A DC vaccine pulsed with a tumor lysate has the ability to generate abundant tumor-specific T cells that can kill tumor cells through the secretion of IFN-γ or lytic granules, and in both preclinical studies and clinical trials^97–101^, DC vaccines have shown impressive results in GBM. The first results from a large phase III clinical trial of an autologous DC vaccine in GBM showed extended survival^102^. Through sample analysis, the amount of TILs was shown to increase in the TME, and this change was recognized as the therapeutic mechanism. With the increase in TILs, the expression of an immune checkpoint molecule simultaneously increased. Recently, several clinical trials have combined DC vaccines and ICIs to improve treatment effects to show promising early results.

In GBM vaccine therapy, GBM stem-like cells (GSCs) and their specific antigens have attracted considerable attention. GSCs and the CD133 stem cell-specific marker drive tumorigenesis and contribute to genotoxic therapy resistance, diffuse infiltrative invasion, and immunosuppression, which are key factors for the incurability of GBM. Previous studies showed that antigen/DC vaccines targeting GSCs, such as AC133 × CD3 bsAb^103^, Sox2 peptides^104^, and GCS specific-antigen pulsed DC vaccines^105^, were capable of inducing T cell immune response, promoting T cell proliferation, and infiltrating into GBM tissue. Thus, it presents promising prospects and better benefits when combined with ICIs.

In addition to glioma vaccination, adoptive cell therapy, such as CAR-T cell therapy, has been investigated given its success in B-cell lymphomas and leukemias^106^. However, in solid tumors, CAR-T cell activity can still be inhibited by the immunosuppressive TME^107^. Therefore, ICIs can assist CAR-T cells in a hostile TME. This help can be given *via* combination administration with ICIs. Clinical trials evaluating CAR-T cell monotherapy in GBM have been conducted, but there have not been any trials studying combination with ICIs. Because CAR-T cells and ICIs are currently the two most promising immuno-oncology approaches, it will be interesting to see how they converge either through combination therapy or genetic engineering.

Viral therapy, while initially designed as a mechanism of gene delivery to increase tumor cell susceptibility to chemotherapy, is now recognized as a form of immunotherapy. Infection of tumor cells with a virus activates the innate immune system, leading to cytokine release and tumor cell lysis. This response promotes the generation of an adaptive immune response to new tumor antigens and potentially the development of a long-term immunotherapeutic effects^108^. While no proven survival benefit has been found, the excitement surrounding this therapy is largely driven by the population of long-term survivors^109^. Several viral therapies, including a replication-defective adenovirus (ASPECT)^110^, a nonlytic retrovirus expressing cytosine deaminase (Toca5)^111^, replication-competent HSV1 (G207), parvovirus (ParvOryx01), and adenovirus (DNX-2401), have been studied in clinical trials, which reported GBM patients with varying responses. The ongoing study of most of these viruses now includes assessing the safety of combining virus delivery with checkpoint inhibition.

## Conclusions

To date, several co-inhibitory immune checkpoint pathways have been identified, and the current list of co-inhibitory receptor pathways has expanded from only the CTLA4 and PD1 pathways to include the LAG-3, Tim-3, TIGIT, and SIRP pathways, which involve innate and adaptive immunities. ICIs have revolutionized the field of cancer immunotherapy. However, not all modes of action are completely understood, and many clinical trials are ongoing to evaluate the safety, tolerability, and efficacy of ICIs and combination therapies. There are still many obstacles to the success of immunotherapies, including the highly immunosuppressive nature of GBM, the lack of biomarkers predicting efficacy, the need to determine the optimal sequence for combination therapy, and the occurrence of severe side effects.

Overall, from the experience accumulated so far, the establishment of a dynamic predictive model and the development of mechanism-driven combination therapies for appropriate patients appear to be the most hopeful advances in GBM immunotherapy.

## Figures and Tables

**Table 1:** Clinical trial of anti-CTLA4 on GBM^a^

Clin. Trial ID	Disease	Interventions	Status	Phase	Completion data	Result
NCT03460782	GBM, glioma	SOC + ipilim	Avail	Phase I	Feb. 2019	Unknown
NCT02829931	Recurrent HGG	Hypofractionated stereotactic irradiation with nivol, ipilim and bevaciz	Recruit	Phase I	Apr. 2021	Unfinished
NCT03425292	Newly diagnosed HGG	SOC + nivol, ipilim, and bevaciz	Recruit	Phase I	Feb. 2022	Unfinished
NCT03233152	Recurrent GBM	Intra-tumoral ipilim plus intravenous nivol	Recruit	Phase I	Nov. 2019	Unfinished
NCT03430791	Recurrent GBM	TTF, nivol and ipilim	Recruit	Phase II	Aug. 2021	Unfinished
NCT03367715	Newly diagnosed MGMT unmethylated GBM	Nivol, ipilim and short course radiation therapy	Recruit	Phase II	Jan. 2020	Unfinished
NCT02311920	Newly diagnosed GBM or gliosarcoma	Ipilim, nivol, or both in combination with TMZ	Active, not Recruit	Phase I	Nov. 2018	Finished
NCT02017717	GBM	Nivol or nivol in combination with ipilim	Active, not Recruit	Phase III	Apr. 2019	Finished
NCT02794883	GBM	Tremelim and durval	Active, not Recruit	Phase II	Jun. 2020	Unfinished
NCT03707457	Recurrent GBM	Nivol with anti-GITR monoclonal antibody MK-4166, IDO1 inhibitor INCB024360 or ipilim	Recruit	Phase I	Feb. 2024	Unfinished
NCT03422094	Newly diagnosed unmethylated GBM	Personalized neoantigen-based vaccine plus poly-ICLC (NeoVax) combined with ICIs	Recruit	Phase I	Apr. 2019	Unfinished

^a^GBM, glioblastoma; HGG, high grade glioma; SOC, standard of care; ipilim, ipilimumab; nivol, nivolumab; bevaciz, bevacizumab; TTF, tumor treating fields; TMZ, temozolomide; tremelim, tremelimumab; durval, durvalumab; Avail, available; Recruit, recruiting.

**Table 2:** Clinical trials of anti-LAG-3/Tim-3 on GBM

Clin. Trial ID	Disease	Interventions	Status	Phase	Completion data	Result
NCT02658981	GBM, gliosarcoma recurrent brain neoplasm	Anti-LAG-3 alone in combination with nivol	Recruit	Phase I	Dec. 2020	Unfinished
NCT03493932	GBM	Anti-LAG-3 combined with nivol	Recruit	Phase I	Jun. 2021	Unfinished
NCT03961971	GBM, multiforme	Anti-Tim-3 in combination with anti-PD1 and SRS (stereotactic radiosurgery)	Not yet recruit	Phase I	Jun. 2023	Unfinished
